# Rare but relevant: Cannabis use and myocardial infarction

**DOI:** 10.1111/add.70128

**Published:** 2025-07-01

**Authors:** Prianka Padmanathan, Emmert Roberts

**Affiliations:** ^1^ Population Health Sciences University of Bristol Bristol UK; ^2^ National Addiction Centre, Institute of Psychiatry, Psychology and Neuroscience King's College London London UK

**Keywords:** cannabinoids, cannabis, cardiovascular pathology, heart attack, myocardial infarction, rare but relevant

## Abstract

Pre‐clinical research and case reports have linked cannabis use to myocardial infarction (MI) since the 1970s. The association with MI may be specific to certain types and patterns of cannabis use as well as certain consumer characteristics; however, due to limited data availability, meta‐analyses examining the association between cannabis use and MI typically report only broad binary categorisations of use vs. no use. Robust prospective studies that capture the complexities of consumption patterns are required to inform causal inferences. In the meantime, clinicians should be aware of the potential increased risk of myocardial infarction in young healthy patients presenting with chest pain and a recent history of cannabis use. Accurate assessment and documentation of recent cannabis use is also essential to improve future research and identify and monitor interactions with cardiovascular medications.

## WHAT IS THE PROBLEM?

Since the 1970s pre‐clinical research and case reports have identified a possible link between cannabis use and myocardial infarction (MI) [1, 2]. Recent changes in the legal status of both medicinal and recreational cannabis products in some jurisdictions, and associated changes in the prevalence and patterns of cannabis use, have led to greater pharmacovigilance [3, 4]. There is now growing recognition of a vast amount of heterogeneity in types and patterns of cannabis use [5, 6]. This, combined with a lack of robust prospective studies, makes drawing definitive conclusions about the causal link with MI difficult [7, 8]. However, several recent epidemiological studies and systematic reviews have added weight to concerns about an association between certain types and patterns of cannabis use and an increased risk of cardiovascular disease, including MI [9, 10, 11, 12].

## HOW DOES IT PRESENT?

Case reports mainly describe healthy young or middle‐age men presenting with symptoms of an MI within hours of cannabis consumption [1, 13]. Most describe cannabis vaping or smoking, but there have also been case reports linking edible cannabis use to MI [13]. Descriptions of the quantity, potency and pattern of cannabis used are often lacking, but where reported, at least 1 g of cannabis had typically been smoked daily for several years [1]. In the majority of cases, ST‐segment elevation has been identified on electrocardiogram (ECG), but angiographic findings vary considerably [1]. Left anterior descending coronary artery occlusion has been described in many cases, but many patients have also been found to have normal coronary arteries and have not required revascularisation [1, 2, 13]. Observational data also indicates a potentially increased risk of cannabis‐associated MI in men compared with women [9]. However, as the population using cannabis changes over time there may be a demographic shift in those affected.

## HOW COMMON IS IT?

In a previous systematic review that ranked triggers of non‐fatal MI based on Odds Ratios (ORs), self‐reported cannabis smoking in the hour preceding symptoms of MI was included based on a single study of 3882 participants who had a non‐fatal MI, of who 124 reported smoking cannabis in the last year. [14, 15] Cannabis smoking ranked third (OR = 4.8; 95% CI = 2.9–9.5), after cocaine use (OR = 23.7; 95% CI = 8.1–66.3) and eating a heavy meal (OR = 7.0; 95% CI = 0.8–66) [14]. The same study estimated the population attributable fraction (PAF) of non‐fatal MIs acutely attributable to cannabis smoking to be 0.8%, based on a prevalence of cannabis smoking of 0.2% and an assumed causal relationship [14].

More recent meta‐analyses investigating the increased risk of MI associated with cannabis use have produced more conservative estimates [8, 12]. An observational systematic review and meta‐analysis of 64 602 083 participants reported a pooled 29% increased odds (95% CI OR = 0.80–2.08) of MI associated with cannabis use (excluding United States Food and Drug Administration‐approved cannabis drugs), compared to no cannabis use [8]. Although a systematic review and meta‐analysis using multinational cohort data from 1 752 353 participants reported a pooled 25% increased risk [95% CI risk ratio (RR) = 0.91–1.71) associated with cannabis use compared to non‐use [12]. Although neither of these studies report a statistically significant pooled association, the crude dichotomisation of the exposure into cannabis use compared to non‐use potentially obscures important associations with particular types and patterns of cannabis use. Furthermore, many of the included studies are cross‐sectional or retrospective, have short‐term follow‐up and/or rely on self‐reported cannabis use or documentation of use within electronic health records rather than biochemically verified objective measures.

In some regions, co‐use of tobacco is common among people who use cannabis [16] and is an often unaccounted for potential confounder in systematic reviews. A recent large cross‐sectional study of more than 430 000 adults (age 18–74 years) in the United States investigated the risk of MI associated with cannabis use within the general population as well as a subgroup who had never used tobacco [9]. The study found a 25% increased odds of MI associated with self‐reported daily cannabis use in the past 30 days compared to no use in both the general population (adjusted OR = 1.25; 95% CI = 1.07–1.46) and the subgroup who had never used tobacco (adjusted OR = 1.49; 95% CI = 1.03–2.15). [9] The reported overall point prevalence of MI was higher for the group of people self‐reporting daily cannabis for the past 30 days (3.6%; 95% CI = 3.1%–4.1%) than for the group self‐reporting less frequent than daily cannabis in the last 30 days (2.9%; 95% CI = 2.6%–3.3%).

## HOW DOES IT OCCUR?

The precise mechanisms by which cannabis use may increase risk of MI remain under investigation. These mechanisms depend on route of administration, the dose, specific cannabinoid concentrations, pattern of use and the demographic and clinical characteristics of the consumer. However, in general the increased risk of MI is primarily thought to occur via activation of the endogenous endocannabinoid system, particularly cannabinoid receptor 1 (CB1R) activation [3, 17]. CB1R is predominantly found in the central nervous system, but also expressed in the cardiovascular system [3, 17]. It has a role in maintaining haemostasis and regulating the autonomic nervous system. The main psychoactive component of cannabis, Δ‐9‐tetrahydrocannabinolic acid (THC), is a partial agonist of CB1R [7]. The other well‐studied cannabinoid, cannabidiol (CBD), appears to have anti‐inflammatory effects in the body and little direct binding affinity to CB1R [3, 7].

In the short‐term, cannabis use is thought to trigger a myocardial oxygen supply–demand mismatch resulting in myocardial ischaemia. THC can induce tachycardia and greater myocardial oxygen demand through CB1R agonism and its effect on the autonomic nervous system [18, 19, 20]. In addition, cannabis smoking contributes to a reduction in myocardial oxygen supply because of increased blood carboxyhaemoglobin levels [21]. These levels may be even higher then after smoking tobacco and appear to be associated with cardiac damage both acutely and the longer term [21, 22]. Clinical findings described in case reports have indicated that cannabis use may also contribute to a reduction in blood flow by inducing coronary vasospasm [23]. However, the mechanism by which this might occur is unclear [18, 23].

Over the longer term, cannabis use could increase risk of MI through platelet aggregation, atherosclerosis and thrombus formation [24]. This was postulated after CB1R and also cannabinoid receptor 2 (CB2R) were detected on platelet membranes [25], but currently there is insufficient evidence to validate the hypothesis [18, 26]. Nonetheless, the risk of MI among people who use cannabis may be increased longer term through an increased risk of other associated risk factors for MI, such as mental illness [27, 28, 29], visceral adiposity [30], arrhythmia [6], and most importantly, co‐use of tobacco [16].

## WHAT ARE THE IMPLICATIONS FOR CLINICAL ASSESSMENTS?

Although uncertainty remains about the exact nature of the relationship between different types and patterns of cannabis use and MI, clinical awareness of a possible increased risk in young healthy patients complaining of chest pain and a recent cannabis use history is important in ensuring timely diagnoses.

As the evidence base develops, clinicians will require an understanding of the distinct biological and clinical effects of individual cannabinoids to inform clinical assessments. For example, in contrast to the possible increased risk of MI associated with THC, pre‐clinical studies indicate that CBD has potential to reduce the risk of MI by reducing heart rate, blood pressure and inflammation. [17, 26] However, one of the first observational studies to stratify cardiovascular outcomes by the cannabinoid content of cannabis highlights that a cautious approach to CBD is required until there is sufficient clinical evidence to assess its use [6, 26]. The Danish study, which compared 5391 patients with chronic pain who commenced medical cannabis for the first time with matched controls, identified an overall higher risk of new‐onset arrhythmias associated with medical cannabis (180‐day adjusted RR = 2.07; 95% CI = 1.34–2.80). When stratified by cannabinoid content, the risk was noted to also be elevated among patients commencing CBD only [180‐day absolute risk (AR) = 1.0%; 95% CI = 0.5%–1.6%) compared to no medical cannabis use (180‐day AR = 0.4%; 95% CI = 0.2%–0.5%) [6, 26].

Some cannabinoids may also, theoretically, interact with antiplatelet and anticoagulant medications used in the treatment and prevention of myocardial infarctions [31]. For example, CBD may inhibit the conversion of clopidogrel to its active metabolite, leading to subtherapeutic levels [31, 32]. CBD may also influence the absorption of direct‐acting oral anticoagulants such as apixaban and rivaroxaban, potentially resulting in increased exposure and bleeding risk [31]. Although these theoretical interactions are not formally recognised at present [33], asking patients who receive anticoagulants and antiplatelets about cannabis use could facilitate detection and improve current understanding.

Clinicians should also be aware of synthetic cannabinoid receptor agonists (SCRAs), often referred to as K2 and Spice, which are an increasingly popular class of drugs that were initially designed to mimic THC. They have a chemically distinct structure to cannabis, but generally bind to CB1R as full agonists with a much higher affinity and greater potency than THC. As such, they may have a greater potential to trigger an MI than cannabis. Evidence quantifying the risk is currently limited, however, they have also been linked to MI in case reports of mainly young healthy individuals [34]. Notably, they are not detected in routine urine drug screens. It is, therefore, important to explicitly ask about both recent cannabis and SCRA use as part of a drugs history when assessing patients presenting with chest pain.

## WHAT ARE THE TREATMENTS?

To date, no specific treatments have been recommended for cannabis‐related MI. There is ongoing investigation into the use of CB1R inverse agonists to reduce cardiovascular risk factors [35] and CB2R agonists to treat chronic conditions including cardiovascular disease [36, 37]. As the pathophysiological mechanisms are better understood, a role may emerge for the use of existing cardiovascular medications, such as beta‐blockers, for the prevention of MI in the context of certain types and patterns of cannabis use [24]. Accurate assessment and documentation of cannabis use in clinical settings is also required to ascertain the appropriateness of its inclusion as a risk factor in cardiovascular risk prediction tools [38].

A key question from a harm reduction perspective is whether some modes of cannabis administration, such as vaping or edibles, might be safer than others. Different modes of administration produce distinct physiological effects and are often associated with varying patterns of use, which can interact and influence the risk of MI. Compared with cannabis smoking, cannabis vaping is associated with less carbon monoxide exposure [39], but also a greater magnitude of increase in heart rate in certain populations and at certain THC doses [40]. Edible consumption results in greater systemic absorption [7]. The co‐use of tobacco also varies between different modes of cannabis administration. Among past‐year cannabis consumers in the United States and Canada (*n* = 6744), smoked cannabis products were associated with an increased odds of all forms of tobacco co‐use [41]. In contrast, cannabis vaping was associated with an increased odds of use of both cannabis and tobacco within the last year and using both on the same occasion, but not with mixing cannabis and tobacco in the same product. Edible cannabis use was associated with a decreased odds of all three forms of tobacco co‐use. A narrative systematic review, which investigated different modes of administration and risk of MI, reported a consistent association between cannabis smoking and increased risk of MI, but not for other modes of administration (mainly edibles but also vaping) [11]. The lack of an association may, however, have been because of a paucity of sufficiently powered studies [11]. Further investigation is, therefore, required.

## WHAT IS THE PROGNOSIS?

A few studies have investigated the effects of cannabis use on post‐MI health outcomes, with conflicting findings [13, 42, 43, 44]. A parallel has been drawn with the ‘smoker's paradox’, whereby decreased mortality rates associated with cannabis use may be because of cannabis consumers being younger with fewer co‐morbidities and cardiovascular risk factors compared with non‐cannabis consumers [43, 44, 45]. As with other aspects of cannabis‐related MI further nuanced research with rigorously conducted prospective observational studies investigating different types and patterns of cannabis use while adjusting for confounding consumer characteristics is needed.

## AUTHOR CONTRIBUTIONS

**Prianka Padmanathan:** Conceptualization; methodology; validation; investigation; project administration; writing—original draft. **Emmert Roberts:** Conceptualization; methodology; validation; investigation; project administration; writing—review and editing; supervision.

## DECLARATION OF INTERESTS

None.

## Data Availability

Data sharing not applicable to this article as no datasets were generated or analysed during the current study.
